# Critical Aspects of Clinical Trial Design for Novel Cell and Gene Therapies

**DOI:** 10.4061/2011/804041

**Published:** 2011-12-27

**Authors:** Zinovia Kefalopoulou, Iciar Aviles-Olmos, Thomas Foltynie

**Affiliations:** Sobell Department of Motor Neuroscience and Movement Disorders, UCL Institute of Neurology, Queen Square, London WC1N 3BG, UK

## Abstract

Neural cell transplantation and gene therapy have attracted considerable interest as promising therapeutic alternatives for patients with Parkinson's disease (PD). Preclinical and open-label studies have suggested that grafted fetal neural tissue or viral vector gene transfer can achieve considerable biochemical and clinical improvements, whereas subsequent double-blind, placebo-controlled protocols have produced rather more modest and variable results. Detailed evaluation of these discordant findings has highlighted several crucial issues such as patient selection criteria, details surrounding transplantation or gene therapy methodologies, as well as the study designs themselves that ought to be carefully considered in the planning phases of future clinical trials. Beyond the provision of symptomatic efficacy and safety data, it also remains to be identified whether the possibilities offered by stem cell and gene therapy technological advances might translate to meaningful neuroprotection and/or disease-modifying effects or alleviate the nonmotor aspects of PD and thus offer additional benefits beyond those achieved through conventional pharmacotherapy or deep brain stimulation (DBS).

## 1. Introduction

Current antiparkinsonian oral drug therapies, with the dopamine (DA) precursor levodopa (L-dopa) remaining the most effective, allow remarkable symptomatic control over the cardinal motor features of Parkinson's disease (PD) in the first years after diagnosis, by restoring the characteristic nigrostriatal DA deficit. Unfortunately, the pharmacotherapeutic window shrinks over time, and treatment is complicated by the onset of motor fluctuations (“ON/OFF” phenomena) and L-dopa induced dyskinesias (LIDs), while signs such as balance disorders, gait freezing, autonomic disturbances, dementia, or affective changes, refractory to dopaminergic substitution, appear [1–3]. Continuous delivery of the DA agonist apomorphine (subcutaneously) or L-dopa (intraduodenally) and surgical strategies such as deep brain stimulation (DBS) provide relief in advanced PD patients with severe motor complications [4–6]. These therapeutic advances, nevertheless, do not influence the underlying neurodegenerative process and have limited effects on L-dopa nonresponsive clinical manifestations, which are now clearly recognized as causes of major disability in late-stage PD [3, 7, 8]. Hence, there is a pressing demand for innovative approaches. Cell replacement therapies and gene transfer through viral vectors into the degenerated host brain have been investigated as alternatives to surpass the shortcomings of conventional symptomatic treatment in PD. Taken together, evidence accumulated so far has provided promising results and proof-of-principle that therapeutic benefits can be achieved, but also generated several unresolved concerns and limitations regarding both these technologies. In this paper, we discuss a number of critical issues that future clinical trials should clearly address before cell-based and gene therapies are considered as clinically relevant treatment options for PD.

## 2. Choosing the Right Intervention

### 2.1. Cell Replacement Strategies

Since the late 1980s, over 300–400 PD patients worldwide have received transplants of human fetal ventral mesencephalic (VM) tissue, rich in post-mitotic DA neurons. The experience accumulated from open-label studies suggested significant clinical benefits across multiple parameters in patients receiving grafted cells accompanied by a general reduction in pharmacological requirements [9–11]. Two double-blind, placebo-controlled transplantation trials showed variable efficacy of transplants and occurrence of side effects, including “off medication” or “graft induced” dyskinesias (GIDs) [12, 13]. Furthermore, a decade after transplantation, it was observed that the PD pathologic process might propagate from host to grafted cells, as indicated by decrease in staining for the DA transporter (DAT) and the presence of intracellular inclusions identical to Lewy bodies [14–16]. Nevertheless long-term follow-up of one of these trials showed consistent efficacy using both clinical and imaging outcome measures [17].

Retrospective analysis of clinical trials and further basic science research have sought to explore factors explaining variability in results of cell transplantation open-label series and double-blind trials and determine whether the most successful cases, which had L-dopa withdrawn and exhibited major clinical improvement for several years, could be reproducible. A number of technical parameters regarding tissue procurement and preparation, such as the age and number of donor fetuses, graft dissection procedures, storage length and conditions, tissue dissociation before transplantation (into pieces or crude cell suspensions), or the use of ancillary neuroprotective strategies to increase graft survival (i.e., glial-derived neurotrophic factor (GDNF), lazaroids), are likely to have an important role [18–20]. It is therefore highly possible that superior and consistent results can be achieved with further optimization of the graft procedure and overall transplant protocol. It has become evident that future trials should attempt graft purification by minimizing the serotonergic (5-HT) component of the grafted tissue, likely to be involved in the development of GIDs, use surgical procedures that give rise to the optimum distribution of cells over the putamen, and adopt an effective mode of carefully monitored immunosuppression for at least 6–12 months post graft [18, 21]. 

Nevertheless the shortage of embryonic donor tissue, difficulties in standardization of cell material (the age at the time of abortion, exact number of donors per putamen) as well as ethical concerns associated with the procurement of tissue from aborted human fetuses make routine clinical application for this type of cell replacement therapy impractical. Alternative cell sources such as autografts of sympathetic neurons from the adrenal glands or xenograft tissue (porcine mesencephalic embryonic neurons) have been abandoned because of discouraging clinical and graft survival results and significant side effects [22–24]. 

Recent progress in the stem cell field has provided impetus to the cell replacement approach. Stem cells of human origin, potentially able to provide an unlimited supply of standardized DA neurons, may be considered as the most promising future source for a cell-based therapy for PD [25]. Cells with the desired molecular, morphological, and electrophysiological profile of A9 substantia nigra DA neurons can be generated from different stem cell sources in vitro, following various differentiation protocols, and can reverse behavioural deficits after transplantation in animal PD models. Embryonic stem cells (ESCs), neural stem cells (NSCs) isolated from embryonic/fetal or adult brain, pluripotent nonneuronal/mesenchymal stem cells (bone marrow, umbilical cord), or the more recently discovered reprogrammed somatic cells such as induced pluripotent SC (iPS) or directly induced neuronal (iN) cells represent potential alternative sources for transplantable material, each having different advantages and disadvantages when considered for a clinical based application (Table 1) [26–32].

The generation of these cells has highlighted the unresolved issue whether entirely pure DA cell populations will be sufficient, since the nondopaminergic neurons and most probably glial compartments present in the embryonic mesencephalic grafts used so far may be important for the differentiation and function of the grafted DA neurons per se, as well as constituting important cellular factories for creating reparative microenvironment at the host's brain [33]. Irrespective of the cell origin, the methods of their harvest, the number of cells available, the mean number of cells injected and their biochemical characteristics will all have to be standardized and sites certified and inspected to ensure their clinical grade before participation in multicentre trial programmes. 

At the present time fetal VM tissue still remains the best standard against which to test future sources of cells for dopaminergic cell replacement in PD. In this context, a new European multicentre project, called TransEuro, was recently launched to further investigate the optimization of the collection, preparation, and storage of fetal tissue for grafting, the selection of the optimal patient group for grafting, evaluate the cause of GIDs, and confirm the efficacy of the transplant technique.

### 2.2. Gene Therapy Approaches

Based on their functional targets (direct stereotactic injection into striatal, nigral, or subthalamic nucleus (STN) cells), and proposed mechanisms of action, gene therapy approaches can be subdivided into 3 main categories: (a) restoration of DA synthesis capacity (enzyme replacement), (b) basal ganglia circuit modulation, and (c) disease modification/neuroprotection through trophic factors. All of these approaches have identified issues in trial design relevant to future investigations. 

Enzyme-replacement gene therapy in PD aims to provide an advantage over peripherally administered L-dopa through restoration of a more physiological manner of dopamine receptor stimulation and/or reduction in the side effects that accompany prolonged peripheral L-dopa administration. These approaches rely on the use of 1 or more of the key enzymes, namely, tyrosine hydroxylase (TH), aromatic L-amino acid decarboxylase (AADC), and GTP-cyclohydrolase-1 (GCH1) to either replace DA directly (Prosavin), enhance DA synthesis from L-dopa (AADC “prodrug”), or supply continuous DOPA delivery [34]. One concern is that ectopic synthesis of DA in a cellular compartment without vesicular storage and release mechanism for this neurotransmitter could result in multiple detrimental effects, that is, exacerbation of dyskinesias, or even degenerative effects due to elevated cytosolic DA levels [35–37]. Preclinical data and open-label studies have been promising with each of these approaches without evidence of excessive dyskinesia production but beneficial effects have been modest and the dose of vector, rate of vector administration, and the extent of coverage of target tissue remain uncertain. Whether recombinant adeno-associated virus-vesicular monoamine transporter 2 (rAAV-VMAT2) should also be incorporated, that would allow transduced cells to effectively process DA, is unclear [38–44]. 

Basal ganglia circuit modulation via gene therapy attempts to inhibit abnormal activity in the STN by increasing the expression of glutamic acid decarboxylase (GAD) (responsible for GABA synthesis) using an AAV2 vector encoding GAD injected into the STN mimicking the effects of DBS [45], and with the aim of delivering the same effective symptomatic relief as DBS but with lower side effects and with greater convenience through long-term symptom alleviation without the need for battery changes. Results from a phase II randomized, double-blinded, multicenter, placebo-controlled trial showed modest beneficial effects [46]; however long-term efficacy and safety of this manipulation remains to be proven, and the ideal trial, that is, a randomised comparison of GAD gene therapy against STN DBS may not be commercially appealing. 

The overall goal for GDNF and Neurturin (an analogue of GDNF) gene therapy is prevention or slowing of the ongoing degenerative processes, as well as strengthening and supporting the dopaminergic phenotype of endogenous nigrostriatal cells [47–49]. Trophic factor therapy is a disease-modifying approach and therefore has the added advantage over pharmacotherapy that it may theoretically alter the progression of the disease. The use of GDNF as a neuroprotectant/neurorestorative agent has been extensively investigated as a PD therapy [50–52]. Bilateral intra-putaminal injection of AAV2-vector encoding a modified form of Neurturin (CERE-120) did not demonstrate benefit at 12 months although an advantage was seen at 18 months in a double-blind randomized controlled trial [53, 54]. This raised questions regarding the retrograde transport of the vector and highlighted the need for adequate periods of follow-up in these types of trials before breaking the blinding process. A trial of Neurturin concomitantly administered to the putamen and substantia nigra is currently underway. 

There are thus a range of potential gene therapy techniques that may confer benefits to PD patients under certain specific situations. The choice of the optimal intervention may, therefore, be individually tailored to specific patient groups (Table 2).

## 3. Patient Selection 

Several clinical trials have been criticized for suboptimal patient selection [12, 13, 52, 53]. While there are inevitably limitations regarding which patients can be recruited to experimental trials of invasive and irreversible treatments, predetermined inclusion and exclusion criteria have critical impacts on the likelihood of a trial meeting its primary endpoints. 

Published series of PD patients who have received fetal VM transplants indicated that the selection of patients younger than 60 years of age, and having a less severe disease (e.g., ≤ stage 3 Hoehn and Yahr scale), had better results at 12 months [12, 13]. With longer-term follow-up, these differential effects diminished [17]. However, both clinical and animal studies suggest that residual spared circuitry provides an elevated level of trophic support for newly grafted cells to thrive [18, 55], therefore it would follow that severe PD patients and those with widespread degeneration outside the striatal target should probably be excluded (at least initially) from cell and gene therapy trials. Furthermore, it is known that severity of GIDs is related to severity of dopaminergic deficit which also plays a role in the development of LIDs, therefore even though other mechanisms are undoubtedly relevant, patients with severe L-dopa-induced dyskinesias should probably not be recruited to cell therapy trials [2, 56]. 

There is to some extent a balanced judgement that needs to be made. With respect to any therapy with potential disease-modifying effects (cell replacement therapy or neurotrophic factor gene therapy), it seems more sensible to target the disease in its earlier stages. On the other hand, the use of experimental, invasive, irreversible techniques in a group of people with relatively mild symptoms needs consideration, in view of the small but important risks associated with functional neurosurgery, although importantly these risks tend to be less among younger people with less brain atrophy. It is possible that *very* early-stage patients may lack the level of degeneration necessary to stimulate trophic factor release needed to support graft survival and outgrowth, although there is only limited animal data to suggest this [55]. 

The degree of response to L-dopa is clearly of relevance for any dopamine replacement based therapy. PD patients with severe tremor that is nonresponsive to dopamine might, in theory, remain with major disability, despite excellent graft survival and function or cell transfection, because of tremor persistence. Exclusion of subjects without any dopaminergic deficits (SWEDDs) needs to be confirmed through routine F-dopa positron emission tomography (PET) or DAT single-photon emission computed tomography (SPECT) imaging at recruitment [57, 58]. Other debilitating symptoms in PD are also caused by pathological changes in nondopaminergic systems leading to postural instability and gait dysfunction, dementia, and autonomic disturbances. Until it is known how to repair these systems, patients in whom such features are predominant should probably not undergo cell transplantation or gene therapy. 

Continuous DOPA delivery utilizes both vector-derived TH and GCH1 but differs significantly from the Prosavin approach in that it depends on endogenous striatal AADC in the host brain for DA synthesis. This therapy therefore, is likely limited to less-severely impaired patients with sufficient endogenous AADC levels. In contrast, all other gene therapy approaches could be applied to a relatively wide range population provided they exhibit a predominance of DA responsive symptoms. 

In addition to age, disease severity, L-dopa responsiveness, and comorbidity based inclusion/exclusion criteria, the impact of these therapies on important genetic subgroups of patients with predominantly motor deficits needs to be determined. Preoperative genotyping for common genetic variants (e.g., parkin, LRRK2 subgroups) is to be recommended to allow planned subgroup analyses for individuals with Mendelian forms of PD.

## 4. Study Objectives/Endpoints

The primary objective of either cell transplantation or gene transfer paradigms is to demonstrate significant improvement across predetermined clinical parameters in a defined patient group [59]. For most gene therapy programmes the objective remains the demonstration of clinical improvement of specific symptoms, while neurotrophic factor gene therapy and cell therapy aim to be restorative. These divergent aims both require robust measures of longitudinal quantitative assessment. 

The most widely accepted measure of PD severity is the Unified PD Rating Scale (UPDRS-Movement Disorders Society modified version) which can be rated in a practically defined “OFF” condition, that is, after 12 hours since last dose of dopaminergic therapy. Accompanying this scale, quantitative measurements of speed (timed tests) can also add objectivity to the assessment of treatment response over time [60]. However, the “OFF” medication evaluation is a somewhat artificial condition, given that many drugs, including L-dopa, are known to have additional “long duration effects” [61]. Changes of the long-term response to L-dopa patterns per se would be worth considering when comparing pre- and post-intervention “OFF” medication states, particularly in exogenous L-dopa-dependent gene therapy approaches [62]. Furthermore, most patients spend the majority of their time in the “ON” medication state and therefore “OFF” improvements are less relevant to their usual functional independence. In view of the diurnal variation “fluctuations” in PD motor control, common with peripheral L-dopa usage, diary data that summarises duration and severity of “ON,” “OFF,” and dyskinetic time can be an additional demonstration of meaningful improvement [63]. Diary data can however be of variable quality, and technology has been embraced to try and improve the reliability of data obtained [64]. Objective measurement of “On drug” and “Off drug” dyskinesia using validated scales and a consistent protocol allows treatment-related changes to be objectively and accurately quantified [65]. 

It has been suggested that time to reach other disease milestones such as the Hoehn and Yahr scale might be more appropriate simple measures from which to objectively judge disease progression [66], and this might be of use in trials of potentially disease-modifying approaches. Measurement of time to develop axial or cognitive symptoms would be of less value in dopamine replacement approaches. Despite this, given that some nonmotor features of PD can be DA responsive and others not, even the dopamine replacement strategies should collect data and evaluate positive or negative effects of their effects on a range of secondary endpoints including the broader nonmotor aspects of the disease, such as cognition, affective changes, autonomic symptoms, and sleep disorders, by employing validated scales [67]. 

Some functional neurosurgery interventions have chosen a patient oriented scale (PDQ-39) as the primary outcome measure [68]. This assessment, in tandem with physician derived assessments that systematically measure disease severity, ensures that data of robust academic value is also translated into meaningful results as prioritised by patients. Economic analyses, utilising tools such as Quality-Adjusted Life Years (QALYs) or EQ-5D, can allow evaluation of cost-effectiveness over time and should routinely be incorporated into trial design. 

Biomarkers that reflect disease state or rate may also serve as surrogate endpoints in clinical trials. At present, there is no biomarker that is sufficiently well accepted to serve as a primary endpoint in a clinical trial of PD cell or gene therapy. The most relevant technologies available are the dopaminergic imaging modalities, including measuring presynaptic AADC activity using PET (^18^F-dopa) and the labelling of the dopamine transporter (DAT) using SPECT [69, 70]. Such neuroimaging endpoints have some advantages over clinical markers, being more objective, more sensitive, particularly in presymptomatic/early stages, however, may themselves be affected by drugs' symptomatic effects. The inclusion of these measures early in trials of cell or gene therapies does, nevertheless, provide important clues about the biological effects of these treatments. In addition, the data accumulated regarding the imaging and the eventual outcome of the patients will help to define the utility of such imaging methods in future studies. The major disadvantages are their additional expense and the fact that most commonly used imaging tools do not quantify the extrastriatal degenerative processes of PD. 

Long-term follow-up is mandatory within cell replacement or gene therapy trial designs. These approaches should not realistically be expected to induce rapid disease modification in PD. A practical decision needs to be made regarding the duration of follow-up before breaking the blind in double blind trials. Even 12-month blinded follow-up may not be sufficient [53, 54]. Moreover, such invasive treatments must be shown to maintain therapeutic effects and acceptable long-term adverse event profiles on a long-term basis (through open-label extension) before they can be eligible for consideration as therapeutic options, as evidenced already [17]. Indeed, since these treatments are likely to permanently change the biology of the nervous system, lifelong follow-up of all treated patients is advised until substantial experience is accumulated. 

Another important aspect of this work is to demonstrate the survival and maintenance of grafted neurons or the gene transduction efficiency of viral vectors to the desired targeted brain areas. Direct proof of such accomplishments is not possible during the life of a patient. Long-term improvements of motor functions or reductions in dopaminergic medications together with PET imaging findings may be correlated against the survival of the graft and degree of satisfactory gene transfer [12, 13, 17, 18, 53, 71]. Permission for postmortem examination should be discussed and obtained during life to enable routine examination when participants die. The postmortem examination of the grafted brains validates the clinical diagnosis of PD, also directly shows the presence of the heterotopic grafted TH neurons in the striatum, and allows a numerical estimate of the number of surviving neurons (viability) of the striatal volume, reinnervated by DA fibres, with reciprocal synaptic contacts with the host's brain. It also shows the evidence of immune events around the transplanted neurons [13]. In PD cases, postmortem studies have validated the preclinical experiments and the postulated dopaminergic reinnervation of the striatum by grafted neurons [72–74]. Moreover such histopathological studies can determine whether and to which extent grafts are affected by the PD pathologic process and indicate the involved mechanisms [14–16]. They also provide pathological evidence regarding the relationships between transplantation protocols and adverse phenomena such as GIDs [75]. 

Given that the occurrence of GIDs in a significant subset of grafted patients has been a major hurdle for further development of cell-based therapies for PD [12, 13, 76], a priority for future trials is to demonstrate that by refining transplantation protocols GIDs can be prevented. Notwithstanding the several theories proposed, recent experimental data have implied that neuronal composition of grafted tissue, and graft derived 5-HT hyperinnervation in particular, is involved in the pathogenesis of GIDs. High 5-HT to DA neurons and 5-HT transporters (SERT) to DAT ratios, respectively, leading to a dysregulation of DA synaptic levels, seem to be significantly contributing to the development of GIDs, and therefore, this evidence suggests that achieving normal striatal 5-HT/DA and SERT/DAT ratios following transplantation should be necessary to avoid the development of GIDs [77]. According to these data, 11C-DASB PET, a marker of SERT availability, pre- and post-transplantation, alongside with clinical assessment of dyskinesias and histopathological data when available, could serve as a useful functional imaging tool to monitor the optimization of transplantation techniques and patient selection and lead to reproducible and marked clinical improvement while escaping the troublesome complication of GIDs [78].

## 5. Data Analysis 

When designing a clinical trial, the number of participants required to reliably answer the clinical question should be carefully considered. Sample size calculations require estimates of effect size and variance of that effect size with relation to the primary outcome measure [79]. This can of course lead to impossibly high numbers given that, in novel cell or gene therapies, estimates of effect size and variance may be crude with limited precision or may only exist for outcome measures aside from the major variable of interest. Statistical rigour is at risk of being sacrificed in the face of the need to perform novel, clinically relevant, and yet affordable trials. Phase II trials with low power or even pilot trials with limited precision should involve experienced statistical support during protocol development, funding application, ethical review, and recruitment and not simply during data analysis and manuscript preparation. This said, in a disorder such as PD that invariably shows relentless progression, short-term blinded comparisons can be complimented by long-term open label follow-up which together can limit the extent of influence due to placebo effects and observer bias. Choosing a cohort sample size is necessarily a fine balance of logistical and pragmatic/ethical considerations that includes and takes account of experienced statistical advice. The selection of appropriate analytical tests, multivariate models, and thresholds for judging significance emerge from this careful planning.

## 6. Sham Surgery Implementation 

Double-blind placebo-controlled trial designs are the essential tools to establish the clinical efficacy of any intervention by minimising bias or placebo effects. Cell replacement or gene therapies for PD represent invasive techniques, requiring direct surgical access to the site of delivery in the brain. “Sham surgery”, the placebo approach taken in most studies [12, 13, 53], thus, requires an elaborate performance, during which many of the procedures involved in the actual treatment are simulated, often including some invasive element such as burr hole creation. Even though the actual risk is quite low and medically significant complications are rare, these approaches are much more invasive than a typical placebo and the risk of short-term pain, stress, and disability during recovery is virtually certain, and therefore, the use of sham surgery is not without ethical controversy [80]. Protocols that involve “awake surgery” require an elaborate pantomime to maintain blinding such that patients remain uncertain regarding their treatment allocation. The completeness of the blinding procedure should therefore be routinely systematically recorded through patient interview. 

Additional risks emerge in protocols requiring general anaesthesia, immunosuppression therapy, and/or significant irradiation exposure for repeated functional imaging scans during evaluation periods, necessary for maintaining the blinding protocol. It is without doubt that substantial and long-lasting placebo effects can occur in PD trials, which might be even more pronounced when surgical interventions are involved, however, some investigators doubt the scientific rationale for using sham surgery for neurotransplantation or gene transfer studies, in the first place [81, 82]. They argue that PD is known to be a progressive degenerative disease that does not improve in its normal natural history, and most likely any placebo effects will wane on the long term. Given that PD trials often involve subjective rating scales as the primary outcome measure, which are vulnerable not only to placebo effects, but to other forms of bias as well, it is currently difficult to escape the demand for sham surgery controlled trials. Still, the use of double-blind sham-surgery-controlled trials should be justified by sufficient preclinical and open-label evidence and considered once neurobiological and technical parameters are standardized. The ethical integrity of sham surgery implementation depends on the ability of the investigators to inform potential subjects about the procedures and the risks involved. Inevitably proof of efficacy for any of the current approaches will compare effects against “placebo” or sham surgery to enable blinding but given the proven effectiveness of existing proven functional neurosurgery—for example, STN DBS, future trials of novel symptom relieving interventions might require comparison against these “conventional” treatment approaches albeit necessarily in an open label manner.

## 7. Current Challenges and Future Perspectives

The experimental field of restorative neurology continues to advance with implementation of cell replacement or gene-transfer-based approaches to treat patients with PD. Both strategies have generated a consensus demonstrating their capacity for structural and molecular brain modification in the adult brain. The source of tissue/cells remains a major subject under consideration for future clinical trials and it is still unknown which cell type(s) may offer the ideal substrate for a transplantation-based protocol in PD. With respect to gene therapy paradigms, the optimal vector, therapeutic protein, and target site remain to be defined, as does, of course the risk of adverse effects associated with both these techniques. 

The requirements for either cell or gene therapies to become clinically competitive treatments for PD are very high. Relief of motor symptoms can be achieved, to a variable extent, by oral medication supplemented by alternative route drug delivery (e.g., apomorphine injections and intrajejunal L-dopa gel) or DBS for advanced-stage disease. Cell and gene therapy treatments will, therefore, have to demonstrate an advantage either in terms of neuroprotection/repair, or in terms of clinical efficacy or safety over both currently available medical and surgical alternatives. The absence of implanted hardware and no need for recurrent battery changes, percutaneous tubes and pumps may represent an advantage over these options in terms of patient convenience. Currently, the safety, efficacy, and disease-modifying capacity of cell and gene therapies remain to be unequivocally proven, and the commercial costs and necessary surgical expertise for their successful delivery will inevitably limit their availability in many countries. 

Far fewer treatment options address the nonmotor aspects of PD, such as dementia, neuropsychiatric symptoms, autonomic failure, and sleep abnormalities, which dominate the clinical, caregiver, and financial burden in advanced stages of the disease [3, 8]. It is tempting to speculate that restoration of the nigrostriatal DA system might physiologically restore DA innervation to extrastriatal regions and thereby improve deficits related to cortical and brain stem dopamine deficiency or could even have consequences that extend to the nondopaminergic neurons. It is possible that those nonmotor symptoms with a focal subcortical anatomical basis represent further symptoms that may respond to targeted gene therapy, while transplantation of different cell types (e.g., glia) might provide more widespread benefits than are currently contemplated [83, 84]. 

The number of parameters that will conceivably influence the outcome of clinical trials of cell or gene therapies is very high. We are currently at a stage where an “explanatory” approach is required to demonstrate that these interventions can have useful effects in the hands of very experienced teams recruiting highly selected patients. Whether any positive findings can be subsequently reproduced on a “pragmatic” basis with broader patient inclusion criteria, and in anticipation of inevitable technical advances and refinement of surgical parameters will be critical in terms of future widespread adoption of any of these interventions.

##  Conflict of Interests

There is no actual or potential conflict of interests in relation to this paper.

## Figures and Tables

**Table 1:** Comparison of tissue/cell sources for cell replacement strategies in PD.

Type of cell	Availability/expandability/proliferation	Capacity to differentiate into DA phenotypes	Tumor formation potential	Ethical concerns	GID	Major advantage
Fetal VM tissue	+/−	+++	−	+	+	Clinical experience
Embryonic stem cells	+++	++	+	++	?	Expandability and differentiation
Neural stem cells	+	+	+/−	+/−	?	Opportunity of autotransplantation
Mesenchymal somatic stem cells	+	+/−	?	−	?	Opportunity of autotransplantation
Reprogrammed somatic cells	++	++	+	+	?	Easily approached autologous source (e.g., fibroblasts retrieved from skin biopsies)

(?): unknown, (−): none, (+/−): poor, (+): some, (++): readily observable, (+++): extensive.

**Table 2:** Patient-centred comparison of gene therapy interventions for PD.

Gene therapy strategy	Advantages	Disadvantages	Patient target group
Neurotrophic factor delivery	Disease-modifying/neuroprotective potential	Likely to cause structural and functional effects beyond the intended targets	Early-stage PD
Prodrug approach	(i) Striatal DA synthesis dependent on subsequent peripheral administration of L-dopa → modulation of (ii) Therapeutic effect still within control of the clinician	(i) Possibility of accumulation of DA in striatal neurons (ii) Increased endogenous DA production, without increased vesicular dopamine storage, could potentially exacerbate dyskinesia	(i) More advanced-stage PD with motor complications (ii) Patients with inadequate striatal AADC activity
Ectopic DA production	(i) Independent of peripheral L-dopa (ii) Independent of endogenous AADC activity		(i) More advanced-stage PD with motor complications (ii) Patients with inadequate striatal AADC activity
Continuous DOPA delivery strategy	(i) Site specific continuous DA—supply (ii) Independent of peripheral L-dopa	Depends on sufficient endogenous AADC activity	(i) More advanced-stage PD with motor complications (ii) Patients with sufficient striatal AADC activity
Modulation of basal ganglia activity	Proposed to mimic clinical results of DBS, which has established clinical effectiveness	(i) Limited experience (ii) Safety concerns due to bilateral irreversible nature of the technique	Same as DBS
